# Supplementary material for: The Virus-Induced Upregulation of the miR-183/96/182 Cluster and the FoxO Family Protein Members Are Not Required for Efficient Replication of HSV-1
Source: Viruses. 2022 Jul 28;14(8):1661. doi: 10.3390/v14081661 (PMC9414244; doi:10.3390/v14081661)
Supplement: Supplementary file 1 [file viruses-14-01661-s001.zip › Figure S3.pdf]

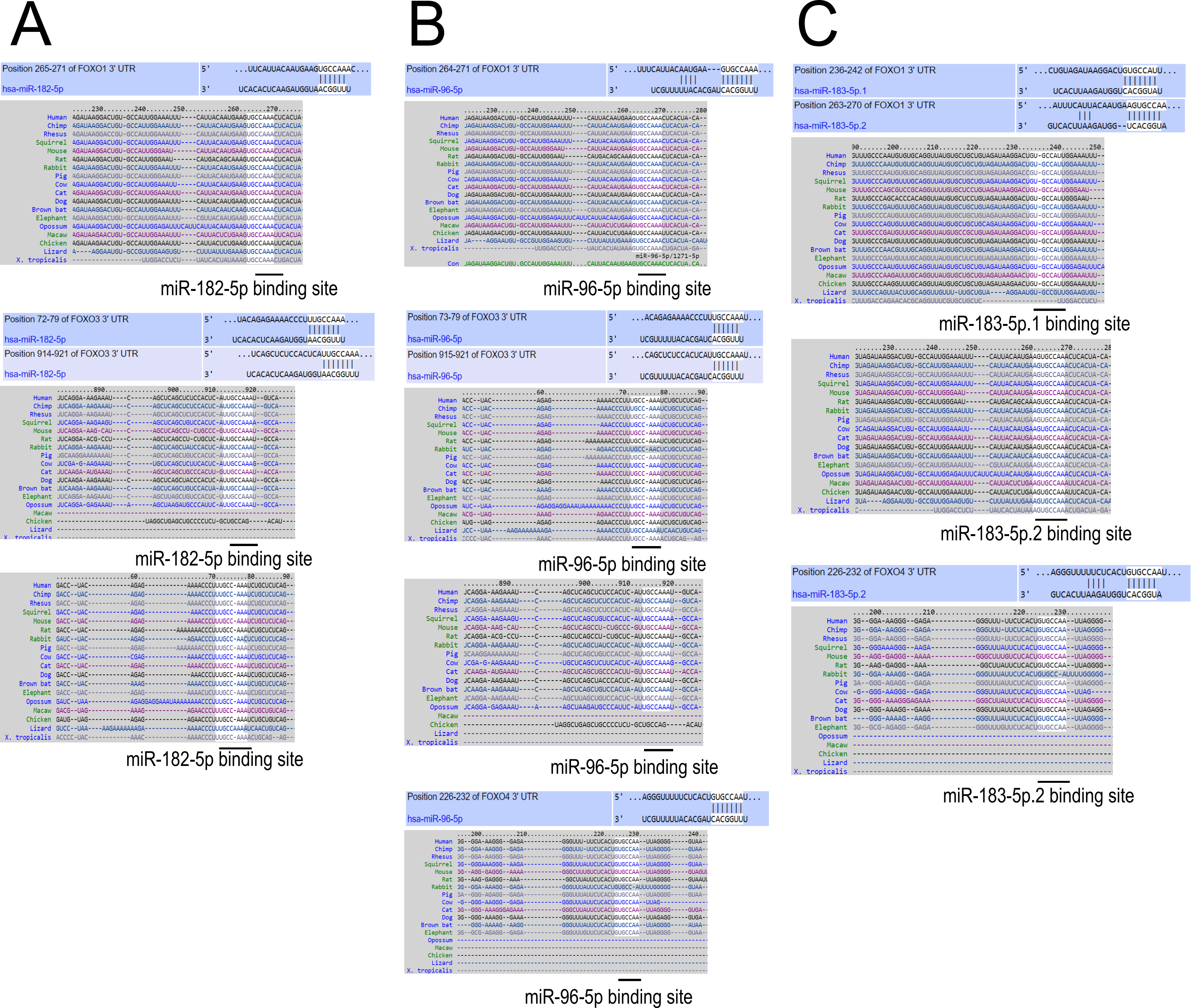
A
Position 265-271 of FOXO1 3' UTR
5' ...UUCAUUACAAUGAAGUGCCAAAC...
hsa-miR-182-5p
3' UCACACUCAAGAUGGUAACGGUUU
miR-182-5p binding site
Position 72-79 of FOXO3 3' UTR
5' ...UACAGAGAAAACCCUUUGCCAAA...
hsa-miR-182-5p
3' UCACACUCAAGAUGGUAACGGUUU
Position 914-921 of FOXO3 3' UTR
5' ...UCAGCUCUCCACUCAUUGCCAAA...
hsa-miR-182-5p
3' UCACACUCAAGAUGGUAACGGUUU
miR-182-5p binding site
miR-182-5p binding site
B
Position 264-271 of FOXO1 3' UTR
5' ...UUUCAUUACAAUGAA---GUGCCAAA...
hsa-miR-96-5p
3' UCGUUUUUACACGAUCACGGUUU
miR-96-5p binding site
Position 73-79 of FOXO3 3' UTR
5' ...ACAGAGAAAACCCUUUGCCAAAU...
hsa-miR-96-5p
3' UCGUUUUUACACGAUCACGGUUU
Position 915-921 of FOXO3 3' UTR
5' ...CAGCUCUCCACUCAUUGCCAAAU...
hsa-miR-96-5p
3' UCGUUUUUACACGAUCACGGUUU
miR-96-5p binding site
miR-96-5p binding site
Position 226-232 of FOXO4 3' UTR
5' ...AGGGUUUUUCUCACUGUGCCAAU...
hsa-miR-96-5p
3' UCGUUUUUACACGAUCACGGUUU
miR-96-5p binding site
C
Position 236-242 of FOXO1 3' UTR
5' ...CUGUAGAUAAGGACUGUGCCAUU...
hsa-miR-183-5p.1
3' UCACUUAAGAUGGUCACGGUAU
Position 263-270 of FOXO1 3' UTR
5' ...AUUUCAUUACAAUGAAGUGCCAA...
hsa-miR-183-5p.2
3' GUCACUUAAGAUGG--UCACGGUA
miR-183-5p.1 binding site
miR-183-5p.2 binding site
Position 226-232 of FOXO4 3' UTR
5' ...AGGGUUUUUCUCACUGUGCCAAU...
hsa-miR-183-5p.2
3' GUCACUUAAGAUGGUCACGGUA
miR-183-5p.2 binding site
Human
Chimp
Rhesus
Squirrel
Mouse
Rat
Rabbit
Pig
Cow
Cat
Dog
Brown bat
Elephant
Opossum
Macaw
Chicken
Lizard
X. tropicalis
