# Supplementary material for: The Virus-Induced Upregulation of the miR-183/96/182 Cluster and the FoxO Family Protein Members Are Not Required for Efficient Replication of HSV-1
Source: Viruses. 2022 Jul 28;14(8):1661. doi: 10.3390/v14081661 (PMC9414244; doi:10.3390/v14081661)
Supplement: Supplementary file 1 [file viruses-14-01661-s001.zip › Supplement figures and figure legends.pdf]

Supplement data figure legends

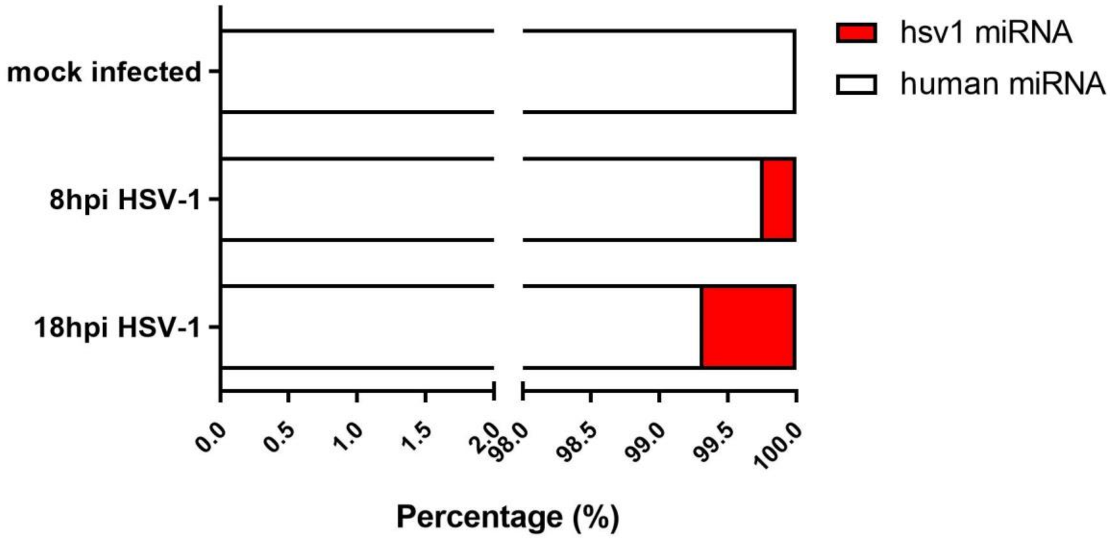

Figure S1: HSV-1 miRNAs represent only a small fraction of miRNAs in infected cells. The percentage of HSV-1 miRNAs in the total number of reads aligned to human and HSV-1 miRNA. The results represent the average of four replicates.

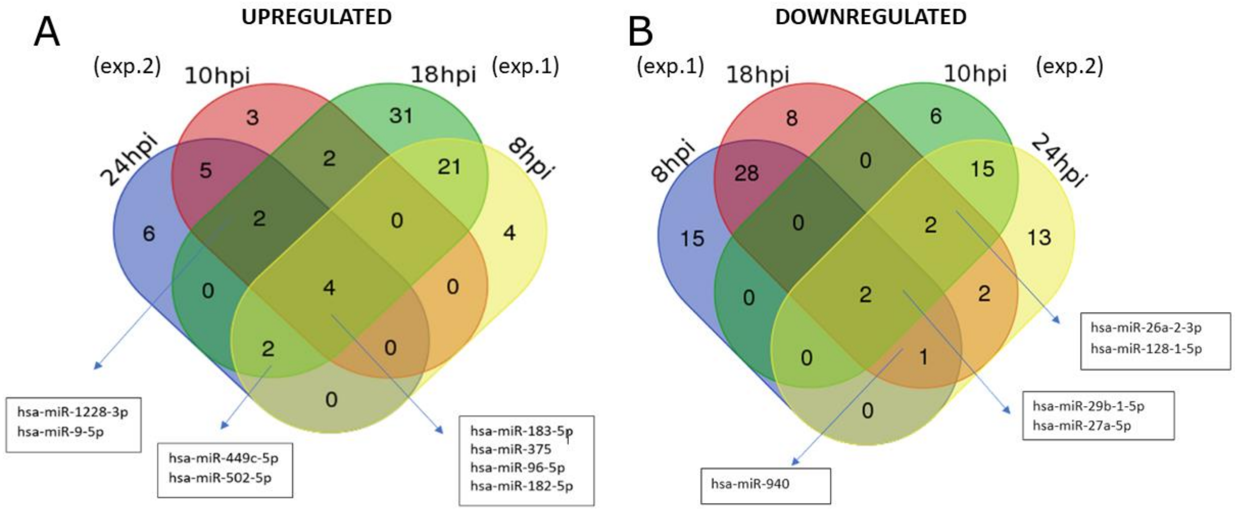

Figure S2: miRNAs found reproducibly deregulated between two data sets. Deregulated miRNAs were extracted from data sets obtained by sequencing samples of HFF cells productively infected with HSV-1 at different time points. Number of miRNAs shared between data sets is shown, and miRNAs found shared between at least three data sets are listed as (A) upregulated or (B) downregulated.

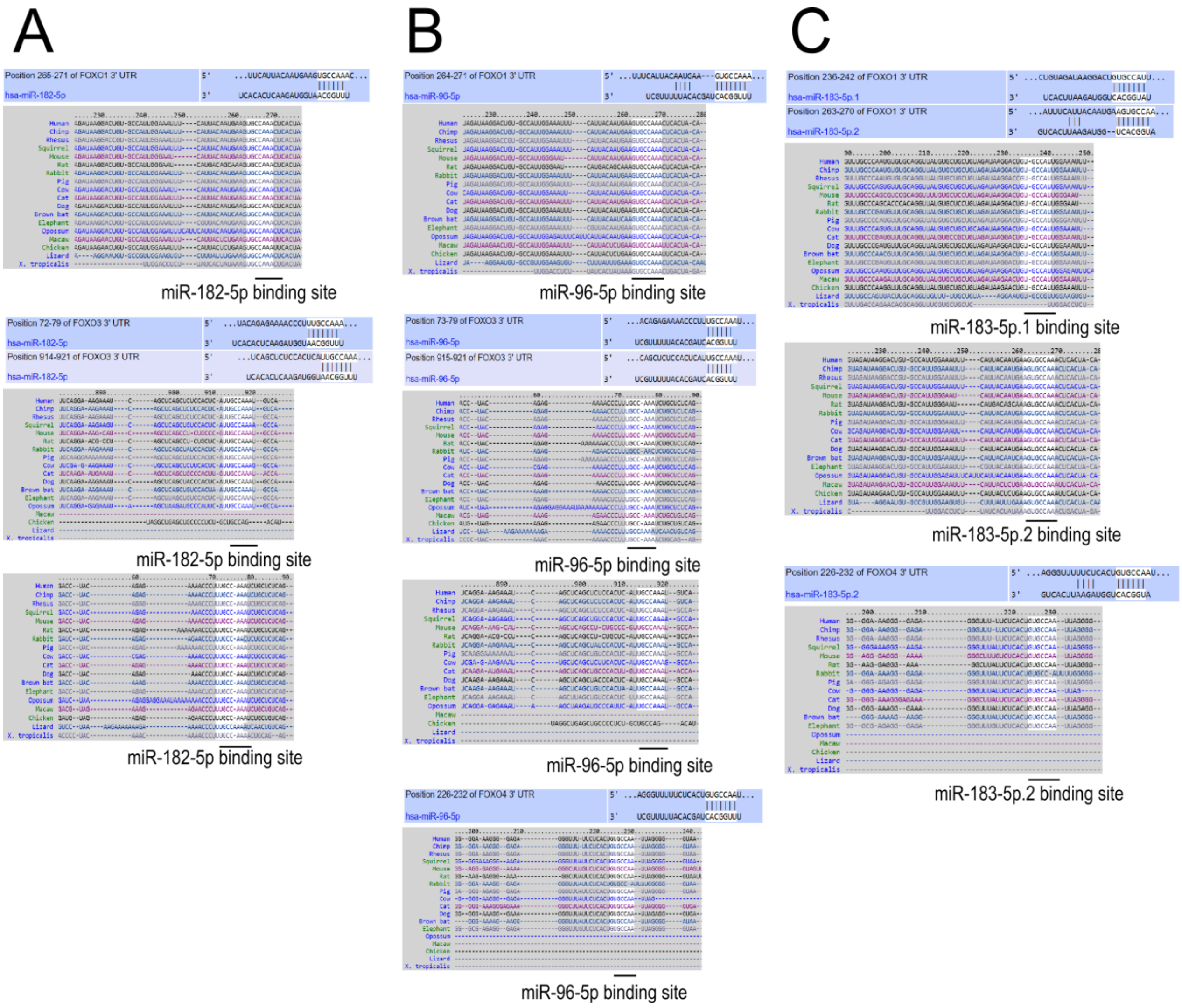

Figure S3: miRNAs miR-182, -96, -183 target 3'UTRs of FoxO genes. TargetScan was used to predict miRNAs: miR-182 (A), miR-96 (B), miR-183 (C) pairing to FoxO targets and their conservation across different species.

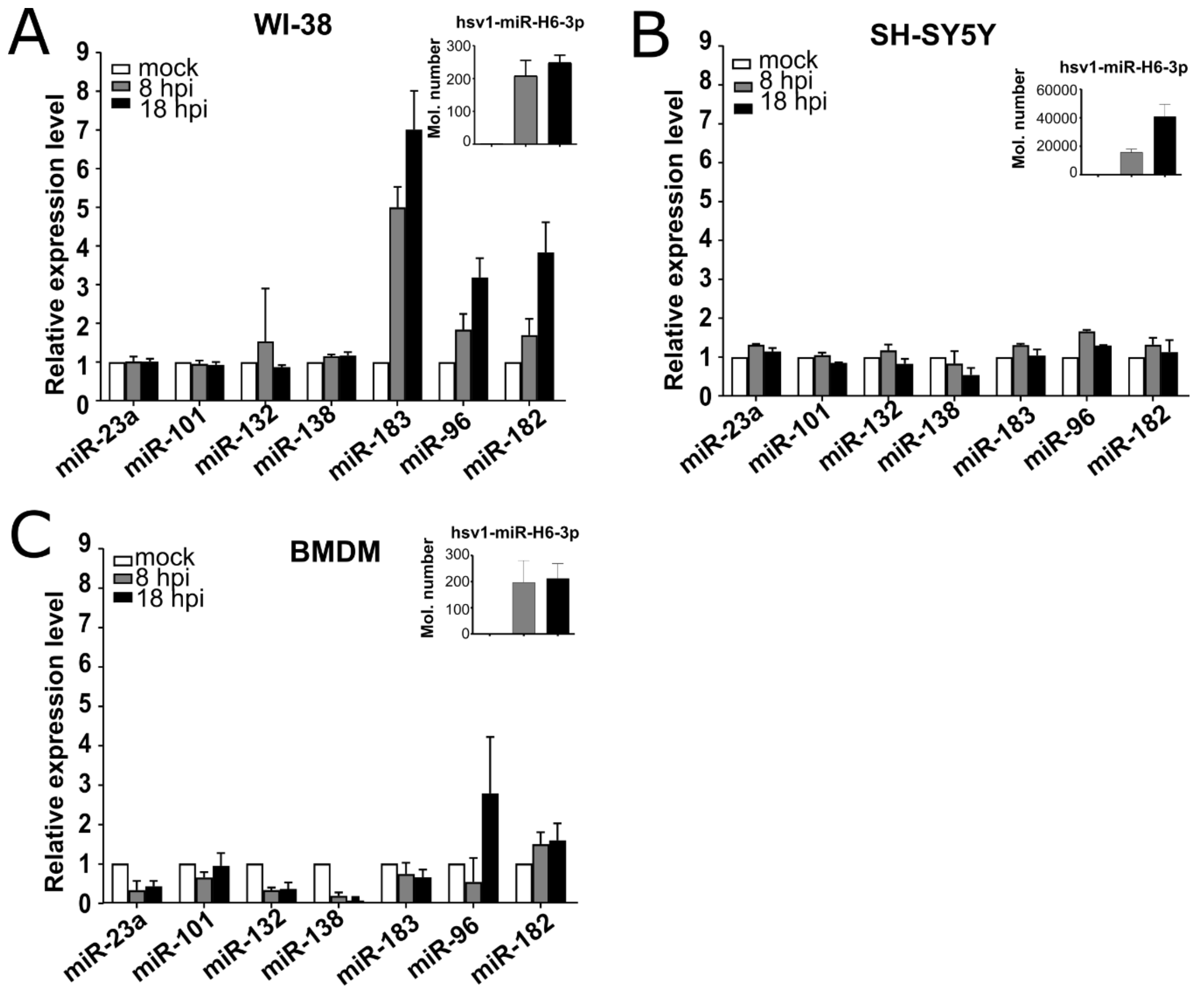

Figure S4: A host miRNA cluster miR-183/96/182 is upregulated in lung fibroblasts. (A, B, and C) Indicated cells (upper part of panels) were infected at an MOI of 10 and total RNA was extracted at indicated time points. The expression levels of miRNAs were determined by RT-qPCR using stem-loop specific assays and normalized to let-7a expression. The expression of vmiR-H6-3p is shown it the upper right corner of the panels.

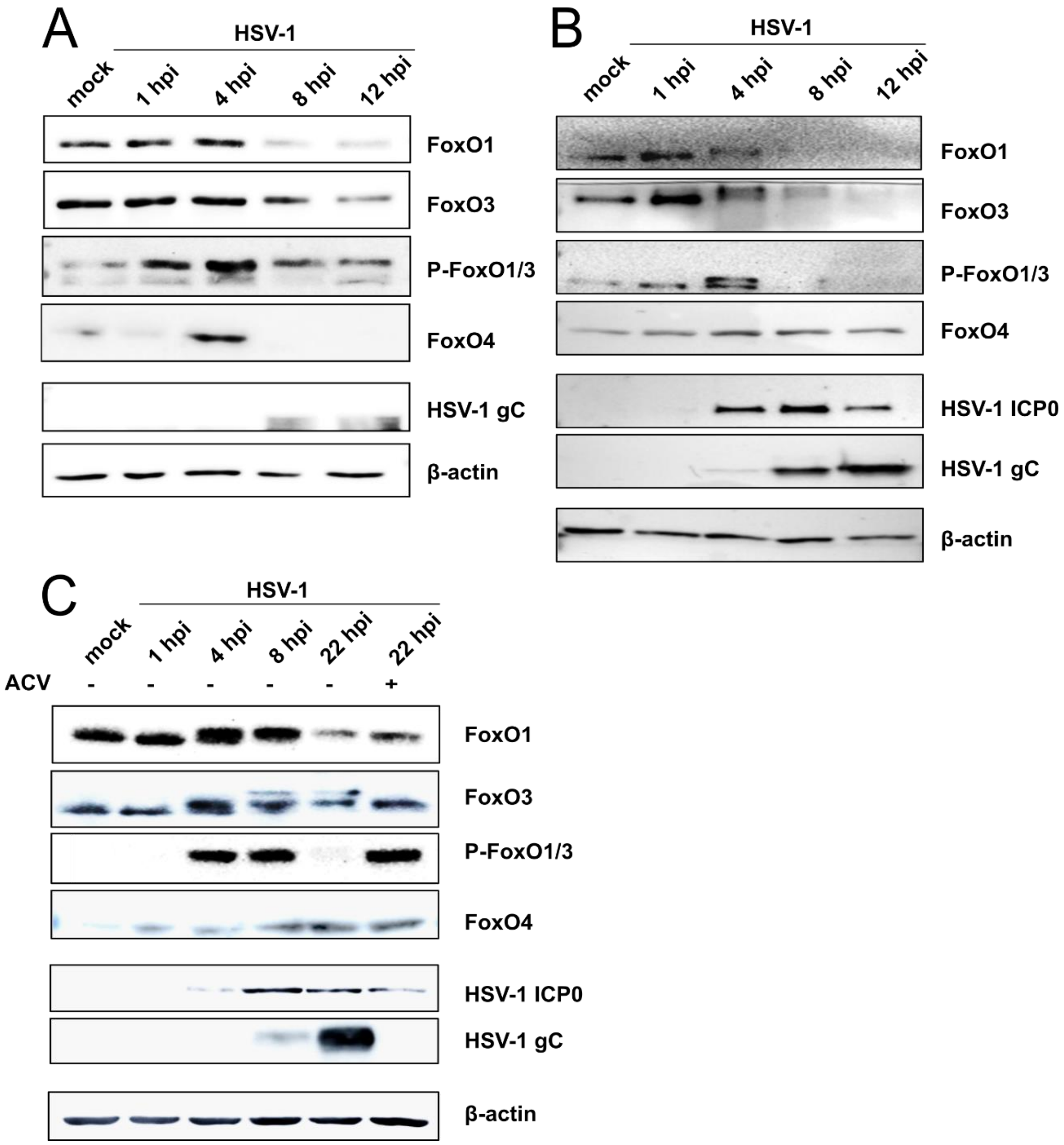

Figure S5: FoxO1 and FoxO3 proteins are upregulated in HEK293, SH-SY5Y and HFF cells. (A) HEK293 cells, (B) SH-SY5Y cells, and (C) HFF cells were mock-infected or infected with HSV-1, where indicated, cells were pretreated with acyclovir (ACV). Cells were collected at indicated time points, and analyzed by western blot. ICP0, immediate-early viral gene, and gC, late viral gene.
