# Supplementary figures and images for: The Virus-Induced Upregulation of the miR-183/96/182 Cluster and the FoxO Family Protein Members Are Not Required for Efficient Replication of HSV-1
Source: Viruses. 2022 Jul 28;14(8):1661. doi: 10.3390/v14081661 (PMC9414244; doi:10.3390/v14081661)

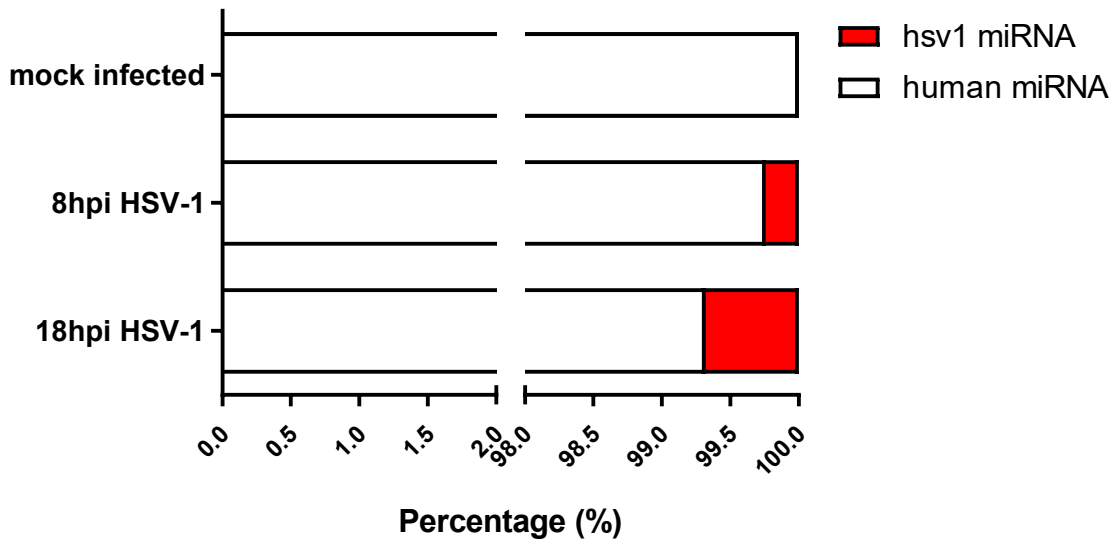

Supplement: Supplementary file 1 [file viruses-14-01661-s001.zip › Figure S1.pdf]

A

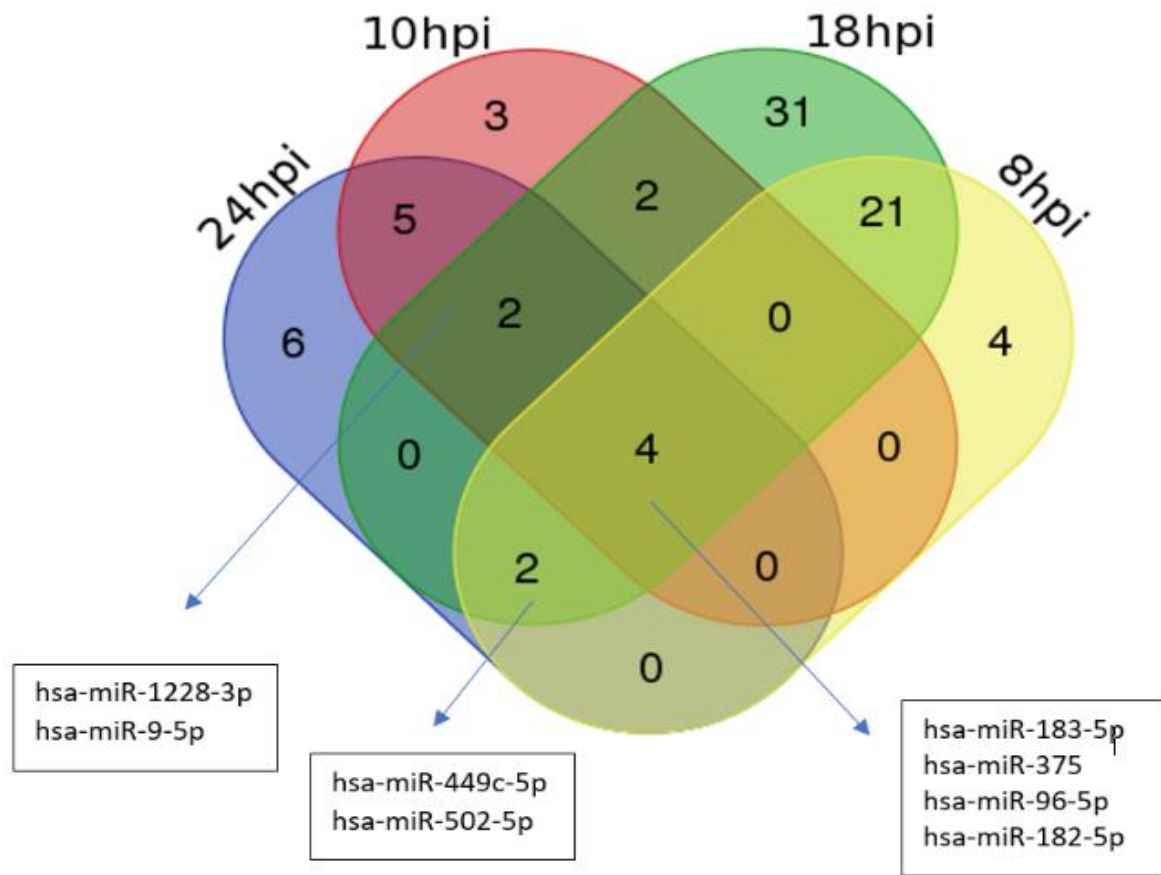

B

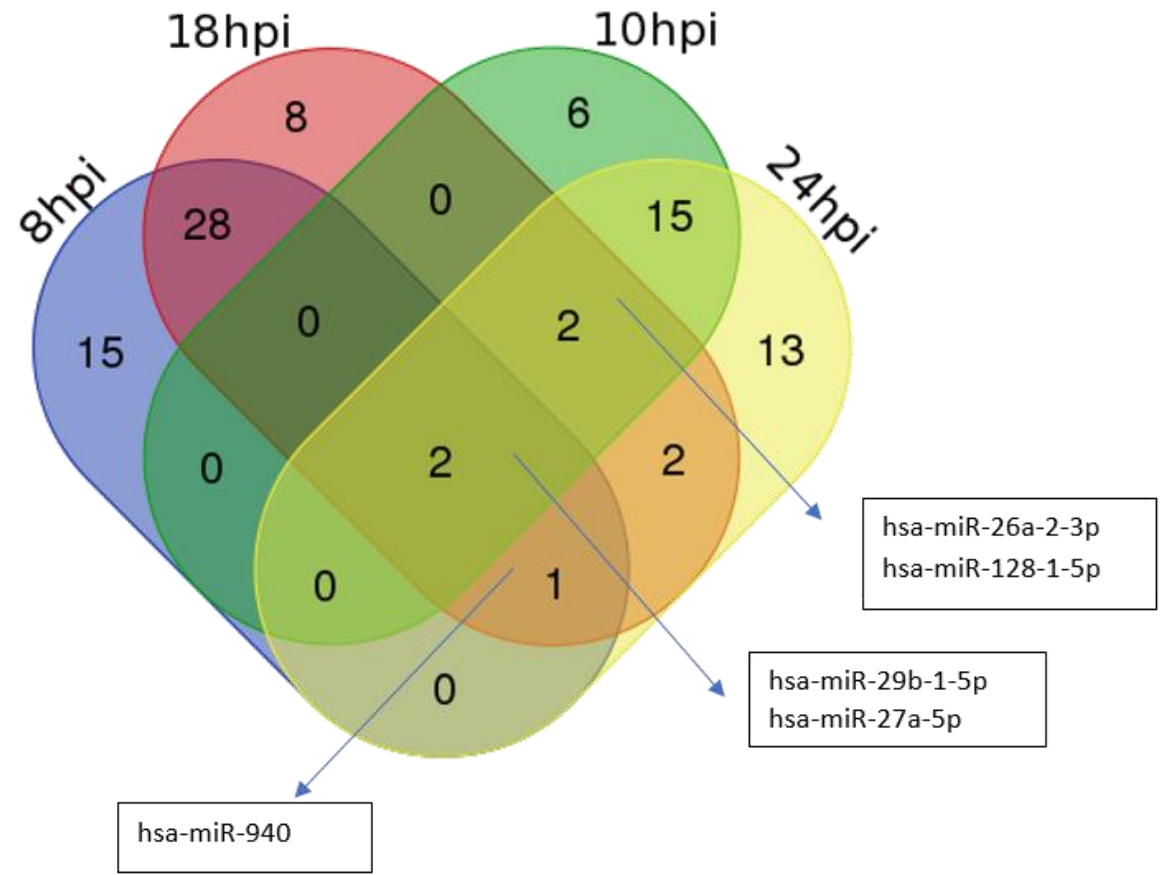

Supplement: Supplementary file 1 [file viruses-14-01661-s001.zip › Figure S2.pdf]

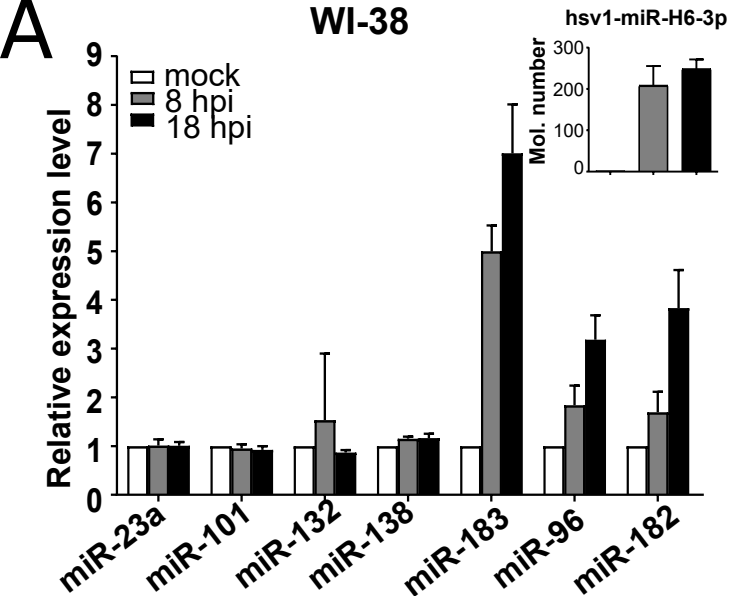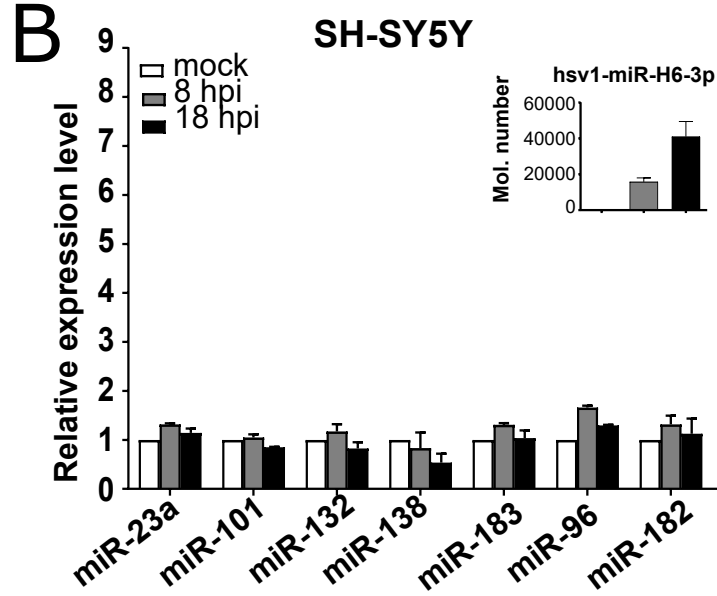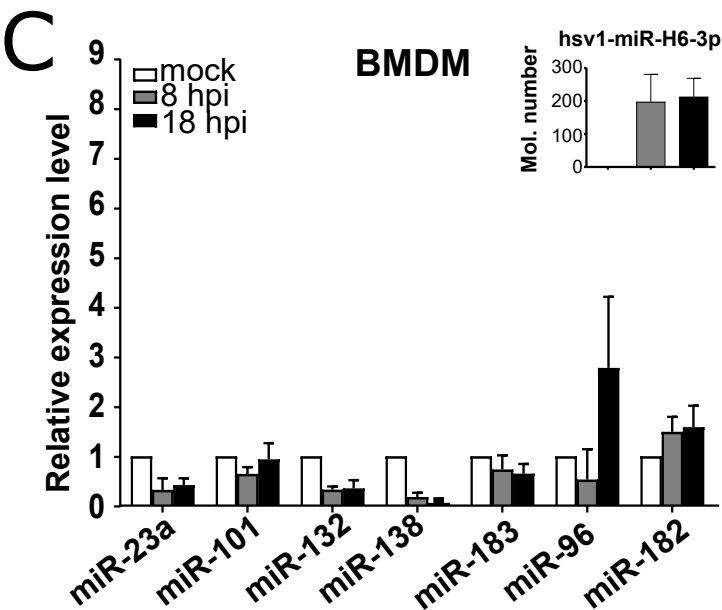

Supplement: Supplementary file 1 [file viruses-14-01661-s001.zip › Figure S4.pdf]

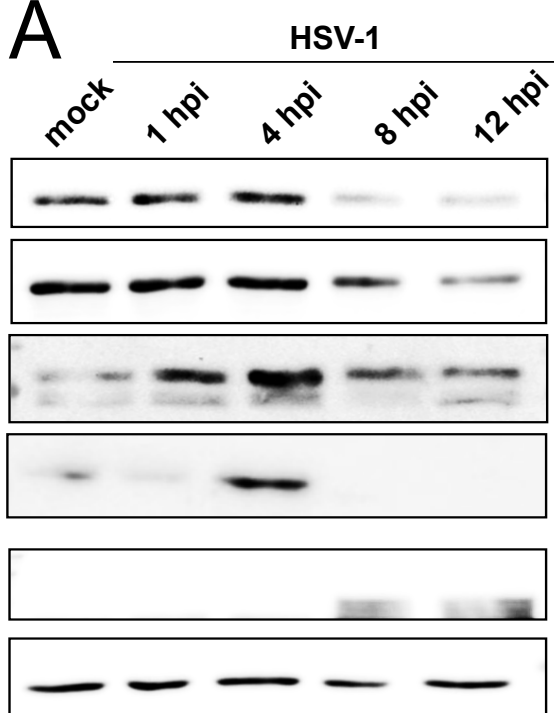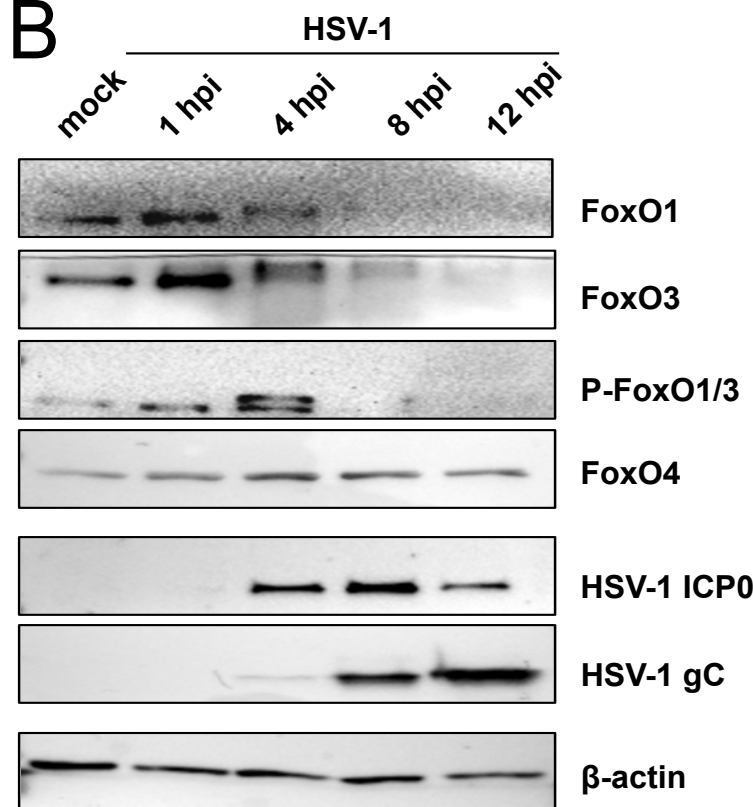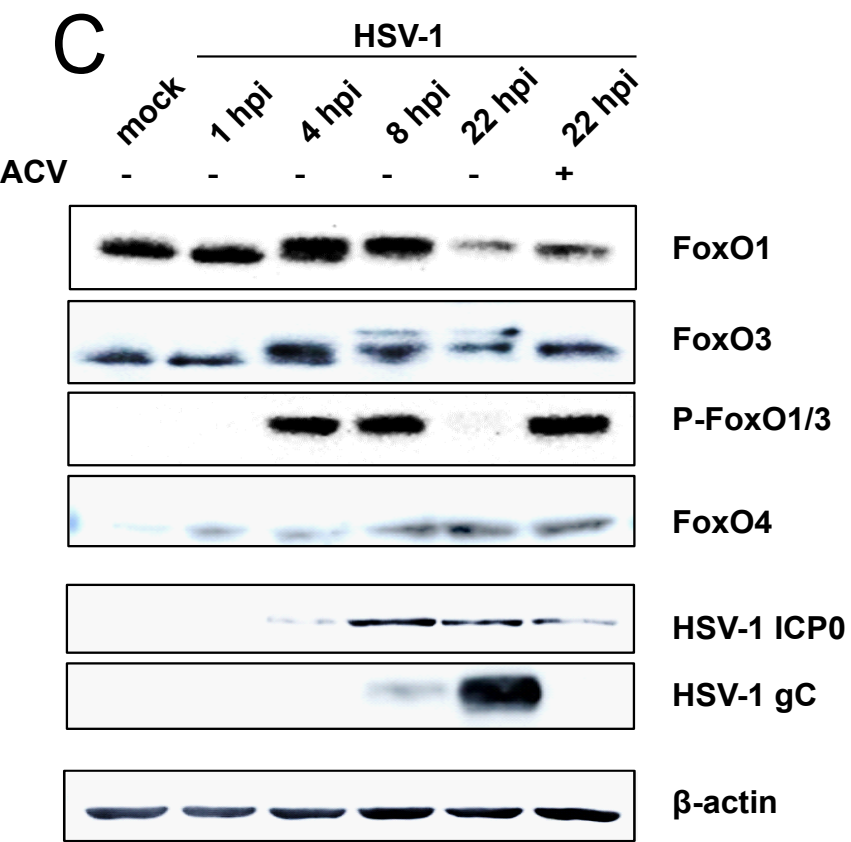

Supplement: Supplementary file 1 [file viruses-14-01661-s001.zip › Figure S5.pdf]
